# Impact of National Drug Price Negotiation policy on the innovation performance of biopharmaceutical industry in China

**DOI:** 10.3389/fpubh.2025.1705030

**Published:** 2025-11-04

**Authors:** Yulu Zhu, Jiazhen Zhu, Xingyuan Gao, Yi Sun, Hongyu Yan, Wenmin Du, Ying Wang, Xin Li

**Affiliations:** ^1^Department of Pharmaceutical Regulatory Science and Pharmacoeconomics, School of Pharmacy, Nanjing Medical University, Nanjing, China; ^2^Department of Health Policy, School of Health Policy and Management, Nanjing Medical University, Nanjing, China; ^3^Department of Infection Management, The First Affiliated Hospital of Soochow University, Suzhou, China; ^4^Department of Pharmacy, The Second People’s Hospital of Changzhou, The Third Affiliated Hospital of Nanjing Medical University, Changzhou, Jiangsu, China; ^5^Center for Global Health, School of Public Health, Nanjing Medical University, Nanjing, China

**Keywords:** National Drug Price Negotiation, biopharmaceutical industry, innovation performance, policy evaluation, difference-in-differences (DID)

## Abstract

**Background:**

The National Drug Price Negotiation (NDPN) policy is a key institutional reform in China that reshaped drug market access rules. While its effects on drug prices and accessibility are well documented, evidence on firm-level innovation performance remains limited. This study evaluates the impact of the 2019 NDPN on the innovation performance of Chinese biopharmaceutical firms.

**Methods:**

We used quarterly panel data from 96 listed biopharmaceutical firms from 2018 Q1 to 2023 Q4. A difference-in-differences (DID) model combined with propensity score matching (PSM-DID) was employed to identify causal effects. Innovation performance was measured by R&D investment, patent application count, and overall growth score, with firm- and time-fixed effects as well as financial controls included. Robustness was assessed through parallel trend and placebo tests.

**Results:**

DID estimates show that firms with NRDL-included drugs significantly increased R&D investment (coefficient = 0.733) and patent application count (coefficient = 0.362). Overall growth score showed no short-term change. PSM-DID confirmed these findings, with R&D investment (coefficient = 0.693) and patent application count (coefficient = 0.272) both significantly improved.

**Conclusion:**

The NDPN policy significantly enhanced firms’ innovation performance by increasing R&D investment and patent applications, though its effect on overall growth remains limited in the short term. These findings provide firm-level evidence that NDPN strengthens innovation incentives in China’s biopharmaceutical industry.

## 1 Introduction

In recent years, as China has accelerated its efforts to promote high-quality industrial development, biopharmaceutical innovation capabilities have continued to strengthen (1). Innovative drugs have become a key direction for breakthrough development in the biopharmaceutical industry (2). As a crucial support for enhancing the core competitiveness of the biopharmaceutical industry and safeguarding public health and well-being, innovative drugs have gradually been integrated into the core agenda of China’s national strategy for building a strong biopharmaceutical nation.

As early as August 2015, the State Council of China issued the “Opinions on Reforming the Review and Approval System for Pharmaceuticals and Medical Devices,” which proposed adjusting the definition of new drugs from “drugs that have not been marketed in China” to “drugs that have not been marketed in China or abroad,” reforming China’s medical product regulatory framework (3). In the same year, the State Council of China issued the “Guiding Opinions on Improving the Centralized Procurement of Drugs in Public Hospitals,” proposing the establishment of an open and transparent price negotiation mechanism for certain patented drugs and exclusively produced drugs. In the same year, the former National Health and Family Planning Commission (NHFPC), the National Development and Reform Commission (NDRC), and the Ministry of Human Resources and Social Security (MOHRSS) launched the first round of National Drug Price Negotiations.

Starting in 2018, the National Healthcare Security Administration (NHSA) began organizing centralized drug negotiations regularly, introducing pharmacoeconomic evaluation as a negotiation tool for the first time (4), and achieving the inclusion of high-cost and high-value drugs in medical insurance through price negotiations, gradually establishing a medical insurance access mechanism that balances affordability and clinical need. Furthermore, to better facilitate the implementation of negotiated drugs, in April 2021, the NHSA and the National Health Commission (NHC) issued the “Guiding Opinions on Establishing and Improving the ‘Dual-Channel’ Management Mechanism for National Medical Insurance Negotiated Drugs,” further promoting the development of innovative drugs.

The main aims of the National Drug Price Negotiation (NDPN) policy are: (1) To achieve “exchanging price for volume” through price negotiation, encouraging bio-pharmaceutical companies to voluntarily lower the prices of innovative medicines for inclusion in the National Reimbursement Drug List (NRDL), thereby ultimately improving the accessibility of negotiated medicines and reducing financial burden of patients; (2) As emphasized in the joint document released by the NHSA and the NHC: to provide genuine support for innovation, to support true innovation, and to encourage differentiated innovation (5). In order to facilitate the shift from generic development to innovation, the NDPN is to guide bio-pharmaceutical companies to increase investment in new drug research and development, and enhance innovation performance.

In November 2019, the 2019 NDPN innovatively introduced a competitive negotiation method, allowing only two drugs with the lowest full-course costs to enter the directory within 2 years, thereby encouraging firms to engage in full competition (6) Compared with previous rounds of negotiations, the 2019 negotiations marked the first attempt to institutionalize and scale up the negotiation framework, significantly enhancing this scientific rigor, procedural standardization, and overall effectiveness (7). This shift has the potential to have a profound impact on the behavior of biopharmaceutical firms. Under such a policy environment, firms are required to fully understand the national-level concept of value-based healthcare and the mechanisms for price formation, and effectively highlight the multi-dimensional value of their products during negotiations to secure satisfactory access prices (8).

Therefore, 2019 was not only a year of institutional transformation for the National Drug Price Negotiation (NDPN) mechanism but also provided a natural analytical window for assessing the impact of medical insurance policies on corporate innovation activities. Building on this policy background, this study uses the 2019 round of negotiations as an exogenous shock event to explore whether and how changes in medical insurance access rules influence the innovation behavior and strategic choices of biopharmaceutical firms in China.

Previous studies have primarily focused on the relationship between China’s National Centralized Volume-Based Procurement (NCVBP) and corporate performance (9), as well as the relationship between corporate innovation (10), relatively few studies have focused on the NDPN policy. For instance, by using panel data and DID models, Li et al. (11) evaluated how China’s NCVBP affected R&D investment of chemical pharmaceutical firms. They compared firms whose drugs were successfully included in the NRDL through price negotiations vs. non-included enterprises and examined heterogeneity by negotiation success rate, price cuts, and firm size. Results showed NCVBP could stimulate R&D, but low negotiation success rates and steep price reductions weaken incentives, particularly for small and medium-sized enterprises. Using panel data and DID estimation, Sun et al. (9) demonstrated that China’s NCDP policy significantly enhanced pharmaceutical firms’ financial performance through cost reduction, market expansion, and increased R&D investment. On the other hand, regarding NDPN policies, most studies have focused on the accessibility (12) and usage frequency (13) of pharmaceuticals in China, with limited attention to the enterprise perspective. Therefore, this study takes the 2019 NDPN as its foundation and employs a difference-in-differences (DID) model to explore the impact of NDPN policy on the innovative performance of biopharmaceutical firms. Compared with previous studies, the potential innovations of this paper may lie in the following aspects:

1. Existing studies have largely focused on the impact of NDPN on drug prices (14), drug accessibility (15), or medical insurance fund expenditures (16), while relatively little attention has been paid to the micro-level effects of such policies on corporate behavior. This study adopts a firm-level innovation perspective to examine how institutional reforms influence biopharmaceutical firms’ R&D investments and innovative outputs, thereby expanding the scope of research on the performance evaluation of medical insurance policies.
2. This paper focuses on the significance of the 2019 NDPN negotiations, which lies in the institutionalization and standardization of the negotiation framework, analyzing its incentive effects on corporate innovation and supplementing micro-level studies of the negotiation system. Additionally, the 2019 reform marked the first full-scale implementation of standardized negotiation procedures. Compared to the 44 and 18 drugs that entered negotiations in 2017 and 2018, respectively, the number of drugs entering negotiations in 2019 reached 150 (17), making this study highly persuasive in terms of both institutional optimization and sample size.
3. This study is based on micro-level data from biopharmaceutical firms, adopts an innovation-focused analytical framework, employs the DID model, further enhanced by a propensity score matching (PSM)-DID double identification strategy to ensure the reliability of the research results. The empirical findings identify the innovation-enhancing effects of the NDPN policy, offering evidence-based insights for assessing the effectiveness and optimization of China’s medical insurance system.

## 2 Materials and methods

### 2.1 DID analysis

The DID model has been widely used to evaluate the effectiveness of policy implementation. Some researchers have utilized the DID model to assess the impact of different policies on corporate financial performance.

The DID model is constructed as follows:


$${\text{Innovation}}_{\mathrm{it}}=\alpha_{0}+\alpha_{1}{\mathrm{DID}}_{\mathrm{it}}+\alpha_{2}{\text{controls}}_{\mathrm{it}}+\mu_{i}+\lambda_{t}+\varepsilon_{\mathrm{it}}$$

The dependent variable Innovation*_it_* is the dependent variable of the model, representing the innovation performance of firm *i* in quarter *t*. The core explanatory variable DID_it_ is a dummy variable assigned based on the NDPN policy document. If the firm belongs to the treatment group and participated in the 2019 NDPN, it is equal to 1; otherwise, it is 0. *α*_1_ is the coefficient of interest, indicating the effect of the NDPN policy on the innovation performance of pharmaceutical firms. If α_1_is positive, it indicates that the implementation of the NDPN policy has improved the innovation performance of biopharmaceutical firms; conversely, a negative value indicates an inhibitory effect. Controls*_it_* is a set of control variables that may influence the financial performance of biopharmaceutical firms. *μ_i_* and *λ_t_* represent the firm-level and quarter-level fixed effects, respectively. *ε_it_* is the random error term.

### 2.2 Data sources and sample selection

The NDPN in 2019 involved 150 negotiated drugs and 70 bio-pharmaceutical firms (18), offering a solid quasi-natural experiment background for this study. Based on the availability and accessibility of data sources, this study selected listed firms in the biopharmaceutical industry on the Shanghai and Shenzhen stock exchanges as the research sample. The financial data of listed firms were obtained from the China Stock Market & Accounting Research (CSMAR) Database, which is one of the most widely used databases in China for empirical research, containing comprehensive financial and market data on over 5,000 listed firms in the Shanghai and Shenzhen stock exchanges, including data on more than 300 firms related to the pharmaceutical and biopharmaceutical manufacturing firms, and the data related to national medical insurance negotiations were mainly obtained from the negotiation results published on the official website of NHSA. Following the methodology of Sun et al. (9), we applied the following exclusion criteria: (1) firms marked as ST (special treatment due to abnormal financial conditions) or ST* (delisting risk warning) in the CSMAR database; (2) firms with significant data missing or abnormal data; (3) the firms that made their initial public offering following the 2019 NDPN. After applying these filters, a total of 96 firms were ultimately included in this study. The treatment group consists of 8 bio-pharmaceutical firms whose products were successfully included in the NRDL through the 2019 national medical insurance price negotiations and the control group consists of 88 bio-pharmaceutical firms that had no products successfully included in the NRDL through price negotiations during 2017–2023 (including 30 firms that applied but were unsuccessful, 28 firms eligible but did not apply, and 30 firms producing only generic drugs and not eligible for participation).

### 2.3 Variable selection

1. Following the studies of Sun et al. (9) and Li et al. (11), the dependent variables in this paper include: R&D investment of biopharmaceutical firms, the number of patent applications, used as a proxy for R&D output, and the overall growth score of biopharmaceutical firms. Compared with previous studies, this approach more reasonably and comprehensively reflects the impact of NDPN policy on corporate innovation.
2. Based on prior research on the impact of national medical insurance policies on enterprises by Sun et al. (9), this study selects the following five firm-level characteristics as control variables:

① Transaction cost (tc), ratio of selling expenses to operating revenue; ② Debt-to-asset ratio (dar), approximately equal to the ratio of total liabilities to total assets; ③ Return on assets (roa), approximately equal to the ratio of net profit to total assets; ④ Book-to-market ratio (bm), approximately equal to the ratio of book value to market value; ⑤ Ownership concentration (oc), defined as the sum of shareholding proportions of the top ten shareholders. These variables and data were obtained from the CSMAR database. Table 1 lists the abbreviations, definitions, and descriptive statistics for all variables.

**Table 1:** Abbreviations, definitions, and descriptive statistics of variables.

Variable Type	Variable	Abbreviation	Definition	Mean	Standard deviation
Dependent variable	Research & development investment	rdi	Research and development cost expenditure (unit: 100 million yuan)	0.554	1.126
	Patent application count	pac	Total number of patent applications filed with CNIPA (China National Intellectual Property Administration) and other agencies during the observation period	5.115	14.116
	Overall growth Score	ogs	Average of growth rates of total assets, net profit, operating income, and net operating cash flow	0.629	12.152
Explanatory variables	DID	did	National drug price negotiation policy dummy variable	0.056	0.229
Control variables	Transaction cost	tc	Selling expenses/operating revenue	0.240	0.198
	Debt to asset ratio	dar	Total liabilities/total assets	0.319	0.170
	Return on assets	roa	Net profit/total assets	0.043	0.046
	Book-to-market ratio	bm	Book value/market value	0.561	0.233
	Ownership concentration	oc	Total shareholding percentage of the top 10 shareholders	52.694	13.100

## 3 Research results

### 3.1 Main DID regression results

Table 2 presents the regression results of the impact of NDPN policy on the innovation performance of biopharmaceutical firms. Model (1) includes firm-level and quarter-level fixed effects, while Model (2) builds on Model (1) by incorporating a set of control variables.

**Table 2:** Impact of the biopharmaceutical negotiation policy on innovation performance: main regression results.

Variable	rdi		pac		ogs	
	Model (1)	Model (2)	Model (1)	Model (2)	Model (1)	Model (2)
did	0.739***	0.733***	0.349***	0.362***	0.743	0.340
	(9.177)	(9.307)	(2.928)	(3.089)	(0.661)	(0.296)
tc		0.064		0.649***		−1.002
		(0.687)		(5.404)		(−0.746)
dar		0.156		0.836***		−3.133*
		(0.825)		(3.131)		(−1.904)
roa		2.000***		5.726***		11.045
		(6.257)		(10.249)		(1.620)
bm		0.466***		−0.217		2.644**
		(5.578)		(−1.567)		(2.167)
oc		−0.016***		0.009***		−0.004
		(−6.505)		(2.646)		(−0.178)
Constant	0.513***	0.958***	0.296***	−0.671***	0.262	0.079
	(43.362)	(5.849)	(5.441)	(−2.762)	(0.211)	(0.040)
Enterprise fixed effects	YES	YES	YES	YES	YES	YES
Quarter fixed effects	YES	YES	YES	YES	YES	YES

The data in parentheses are statistics, and ***, **, and * indicate significance at the 1, 5, and 10% levels, respectively.

The results show that the coefficient of the DID variable is 0.739 (in 100 million RMB) when R&D investment (rdi) is used as the dependent variable, and is statistically significant at the 1% level. This indicates that firms participating in the NDPN policy exhibit substantially higher R&D expenditure than non-participating firms, demonstrating the policy’s strong incentive effect. The DID coefficient in Model (2) remains statistically significant at the 1% level, validating the robustness of the policy’s impact.

In the model with patent application count (pac) as the dependent variable, the DID coefficient is positive and remains statistically significant, indicating that the healthcare negotiation policy has a direct incentivizing effect on the patent output of firms. The DID regression coefficient of the overall growth score (ogs) is not significant, indicating that the short-term effect of NDPN policy on enterprises’ overall growth capacity is not yet evident.

These empirical findings indicate that the NDPN policy primarily exerts its effects in the initial stages of implementation by increasing firms ‘investment in R&D activities and innovation output, while its impact on firm financial growth may exhibit lagged effects. From a theoretical standpoint, NDPN compresses the profit margin of biopharmaceutical firms through price mechanisms, forcing them to enhance their R&D capabilities to maintain competitive advantages (19). In the short term, this pressure is reflected in heightened R&D investment, whereas the transformation of investment into concrete innovation outcomes and enhancements in growth potential generally requires time to materialize. Additionally, factors such as corporate governance structure and firm profitability also influence policy outcomes to some extent, and their mechanisms should be further explored in future research (20).

### 3.2 Robustness test

#### 3.2.1 PSM-DID

Although the DID model used earlier mitigates the endogeneity issue between policy implementation and firm innovation investment to some extent, it does not fully account for potential sample selection bias. Due to significant heterogeneity among firms in terms of size, financial conditions, and governance structures, the likelihood of firms being selected into the NDPN policy is not entirely random. This may lead to systematic pre-treatment differences between the treatment and control groups prior to policy implementation, thereby affecting the validity of DID estimates. To address this issue, this study further introduces the propensity score matching (PSM) method and constructs a PSM-DID model. By matching treatment and control groups with similar characteristics, the PSM-DID model aims to eliminate sample selection bias as much as possible while controlling for unobservable, time-invariant firm heterogeneity. This approach enables a more precise estimation of the net effect of the NDPN policy.

Additionally, to enhance the credibility of causal identification, this study adopts the analytical strategy proposed by Ham and Miratrix (21). After completing PSM, a parallel trend test is conducted on the matched samples. This approach helps mitigate the impact of sample heterogeneity while verifying the validity of the key assumption underlying the DID model, thereby enhancing the robustness and interpretability of the estimation results.

**Figure 1:**
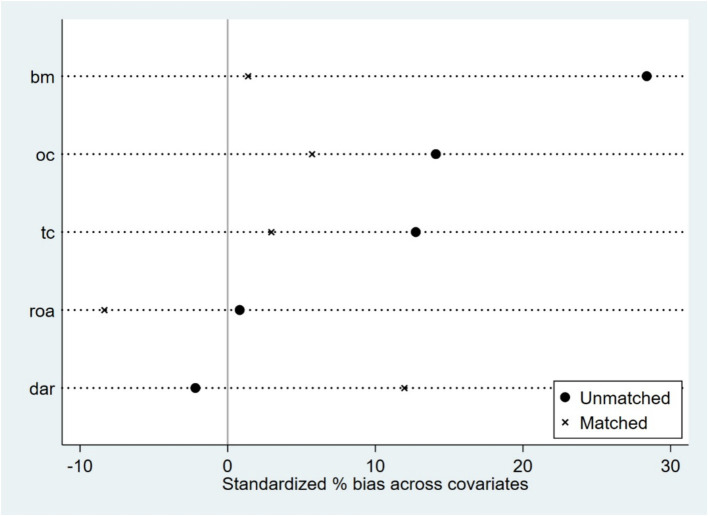
Covariate balance plot.

**Figure 2:**
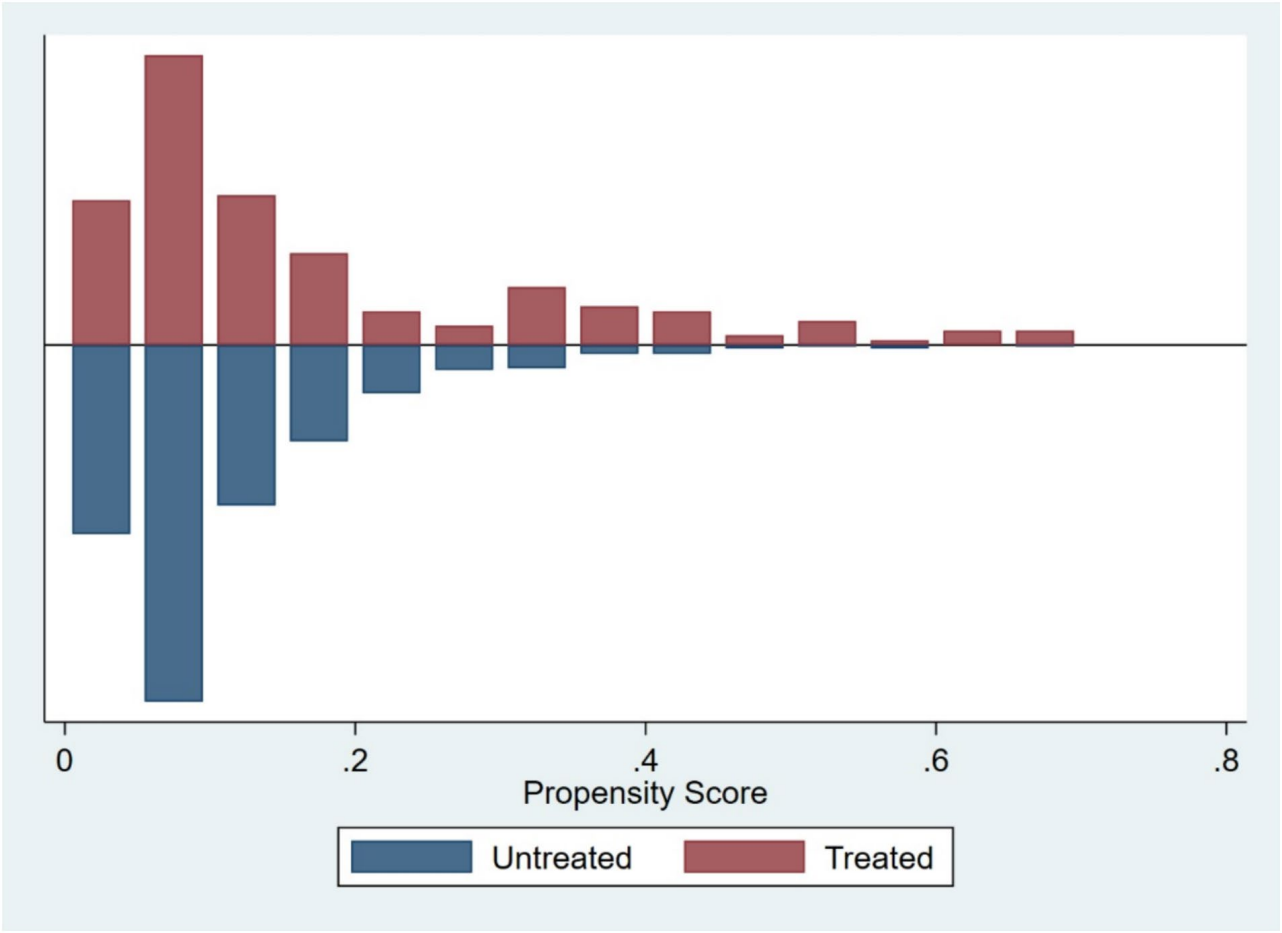
Propensity score distribution chart.

Based on firm characteristics data prior to the policy implementation, propensity scores were estimated using the nearest neighbor matching method (1:4), and a logit regression model was employed to match firms by combining key characteristic variables (tc, dar, roa, bm, oc), with a caliper value of 0.2. Post-matching balance tests indicated (Figure 1; Table 3) that, except for dar, the absolute values of standardized mean differences (SMDs) for all variables fell below the 10% threshold. The mean differences between the treatment and control groups for all variables are statistically insignificant (*p* > 0.05), indicating that sample selection bias has been effectively mitigated. The distribution plot of propensity scores (Figure 2) shows that the treatment group and the control group are highly overlapping, meeting the common support domain requirement.

**Table 3:** Balance test results.

Variable	Unmatched/matched	Mean		Standard deviation (%)	Reduction in deviation (%)
		Treatment group	Control group		
tc	U	0.269	0.248	12.700	76.800
	M	0.269	0.264	2.9	
dar	U	0.329	0.333	−2.200	−446.600
	M	0.329	0.308	12.000	
roa	U	0.042	0.042	0.8	−928.800
	M	0.042	0.045	−8.400	
bm	U	0.745	0.680	28.400	95.200
	M	0.745	0.742	1.4	
oc	U	55.279	53.484	14.1	59.500
	M	55.279	54.552	5.7	

Subsequently, a DID regression analysis was conducted using the matched sample (Table 4). The core results indicate that the policy has a significant promotional effect on R&D investment (rdi) and patent application count (pac), consistent with the direction of the baseline DID results; and overall growth score (ogs) was not significantly affected.

**Table 4:** PSM-DID regression results.

Variable	rdi	pac	ogs
did	0.693***	0.272**	1.030
	(6.570)	(2.270)	(1.205)
Constant	1.393***	−0.744	0.076
	(2.879)	(−1.453)	(0.027)
Enterprise fixed effects	YES	YES	YES
Quarter fixed effects	YES	YES	YES

The data in parentheses are statistics, and *** and ** indicate significance at the 1 and 5% levels, respectively.

These PSM-DID results provide robust support for the findings from the baseline DID model, indicating that the NDPN policy has a stable and significant positive effect on firms’ R&D investment and innovation output, while its impact on broader firm-level financial performance remains inconclusive and warrants further investigation.

#### 3.2.2 Parallel trend test

**Figure 3:**
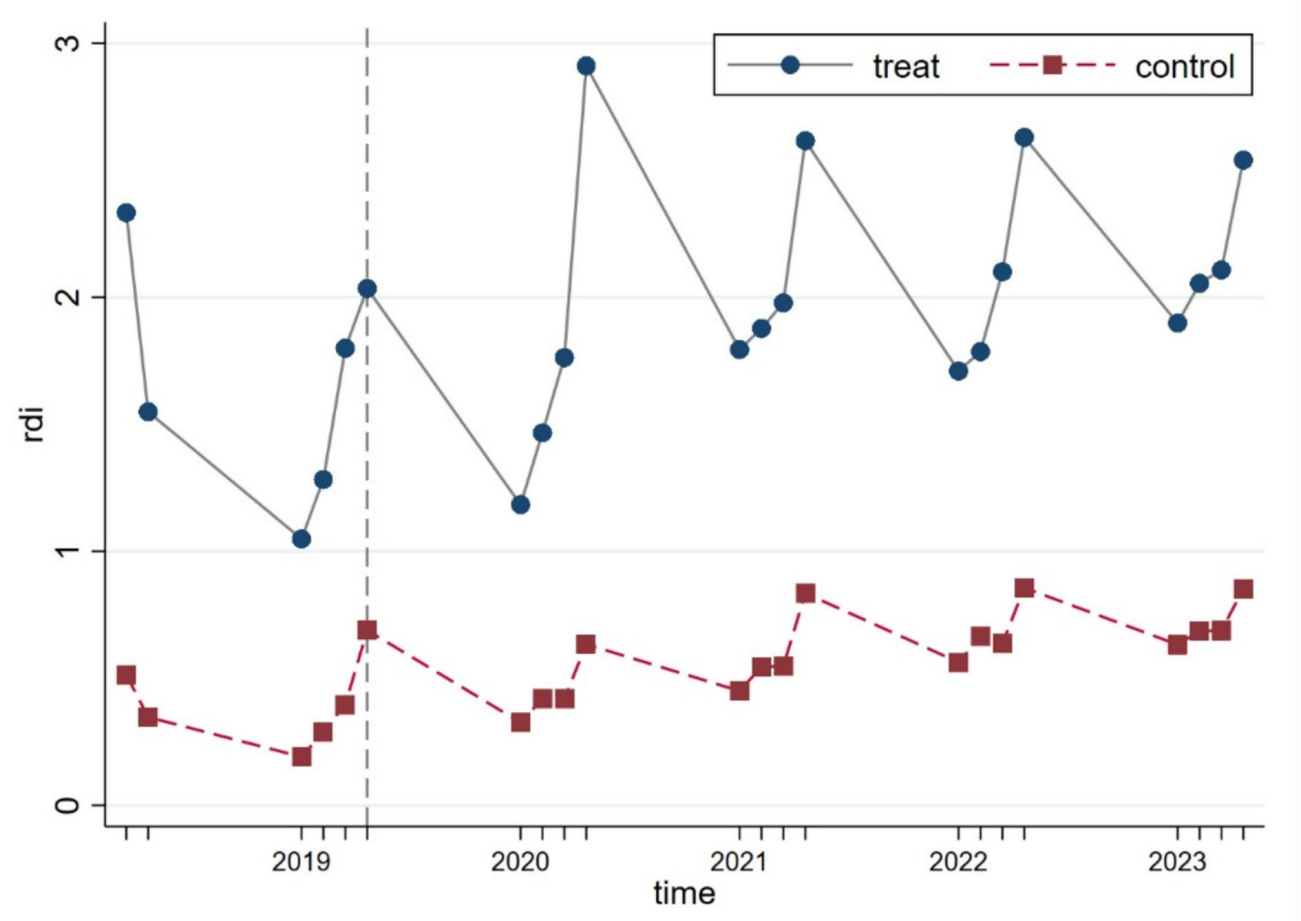
Trends in R&D Investment (rdi) for the treatment and control groups, Q2 2018—Q4 2023.

To verify whether the parallel trend assumption underlying the DID model holds, we conducted a visual inspection using the continuous linear variable R&D investment (rdi). As illustrated in Figure 3, the results show that prior to the policy implementation, the treatment and control groups exhibited similar trajectories, with no significant structural deviations observed, thus supporting the validity of the parallel trend assumption.

For the pac variable, due to the large number of zero values and the presence of extreme outliers in the sample, graphical parallel trend tests are likely to be unreliable. Following the approach in prior literature (22), parallel trend tests were conducted using a regression form.

The constructed model is specified as follows:


$$\begin{array}{l} {\text{Innovation}}_{\mathrm{it}}=\alpha_{0}+\alpha_{1}{\mathrm{DID}}_{\mathrm{it}}^{-7}+\alpha_{2}{\mathrm{DID}}_{\mathrm{it}}^{-6}+\alpha_{3}{\mathrm{DID}}_{\mathrm{it}}^{-5}+\cdots +\\ \alpha_{7}{\mathrm{DID}}_{\mathrm{it}}^{-1}+\alpha_{8}\;{\text{controls}}_{\mathrm{it}}+\mu_{i}+\lambda_{t}+\varepsilon_{\mathrm{it}} \end{array}$$

In this model, we introduce interaction terms between the treatment group and each of the seven quarters prior to policy implementation. These interaction terms are incorporated into a two-way fixed effects model to conduct regression analysis. The seven consecutive quarters prior to policy implementation were used to construct interaction variables between the treatment group and each quarter, which were then jointly included in the regression model to conduct a joint significance test on these interaction terms. If the test results are not significant, it indicates that there are no systematic differences in innovation performance between the treatment group and the control group prior to policy implementation, thus supporting the credibility of the DID model estimates. The regression results of the parallel trends test are reported in Table 5, where DID^−1^ to DID^−7^ represent Pre_1 to Pre_7, respectively.

**Table 5:** Parallel trend test results of pac.

Variable	pac
Pre_1	0.130
Pre_2	−0.213
Pre_3	−0.014
Pre_4	0.670***
Pre_5	0.149
Pre_6	0.222
Pre_7	0.026

The *** indicate significance at the 1% levels, respectively.

For the pac variable, the treatment effects in the pre-policy period were not significant for the majority of cases. This indicates that the dependent variable did not exhibit a significant trend prior to policy implementation, supporting the parallel trends assumption and providing a solid foundation for subsequent DID estimation.

Additionally, the observed significance of Pre_4 may be attributable to an acquisition by a biopharmaceutical firm from the East China region, which took place in 2018. This acquisition, completed in Q4 2018, involved the change of control of a biopharmaceutical firm. Following the change in control, the integration and re-filing of patent resources may have led to a disruption in the number of patent applications, which is reflected in the significant coefficient for Pre_4.

#### 3.2.3 Placebo test

To mitigate the impact of outliers on the research results, this study followed the research methods of Zhao et al. (23) Specifically, the dependent variables were winsorized at the top and bottom 1 and 2.5% quantiles for all continuous variables, and then reintroduced into Model (2) for regression. The regression results are reported in Table 6. The results indicate that the estimated coefficients of the core explanatory variable DID remain significantly positive, supporting the hypothesis that the NDPN policy enhances the innovation performance of biopharmaceutical firms.

**Table 6:** Results of winsorized data.

Winsorizing Level	1 and 99%	2.5 and 97.5%	1 and 99%	2.5 and 97.5%
Variable	rdi		pac	
did	0.729***	0.672***	0.271**	0.224*
	(8.679)	(9.462)	(2.287)	(1.941)
Constant	0.861**	0.762**	−0.860*	−0.803
	(2.230)	(2.307)	(−1.665)	(−1.535)
Enterprise fixed effects	YES	YES	YES	YES
Quarter fixed effects	YES	YES	YES	YES

The data in parentheses are statistics, and ***, **, and * indicate significance at the 1, 5, and 10% levels, respectively.

#### 3.2.4 Sensitivity analysis

Due to the large difference in sample sizes between the treatment group (*N* = 8) and the control group (*N* = 88), concerns regarding the statistical power and generalizability of the findings may arise. To address this issue, this study conducted a sensitivity analysis using a jackknife procedure, where one firm from the treatment group was sequentially excluded, and the regression models were re-run. The results are presented in Table 7. The firms in the treatment group are Firm A to Firm H.

**Table 7:** Sensitivity analysis: jackknife results.

Exclusion Condition	did	
	rdi	pac
Original model (*N* = 8)	0.693***	0.272**
	(6.570)	(2.270)
After Excluding Firm A	0.608***	0.343***
	(5.467)	(2.597)
After Excluding Firm B	0.838***	0.204
	(7.465)	(1.615)
After Excluding Firm C	0.775***	0.444***
	(9.325)	(3.394)
After Excluding Firm D	0.790***	0.291**
	(7.015)	(2.360)
After Excluding Firm E	0.784***	0.293**
	(6.811)	(2.432)
After Excluding Firm F	0.705***	0.226*
	(5.702)	(1.798)
After Excluding Firm G	0.671***	0.292**
	(6.006)	(2.270)
After Excluding Firm H	0.488***	0.226*
	(4.667)	(1.663)

The data in parentheses are statistics, and ***, **, and * indicate significance at the 1, 5, and 10% levels, respectively.

The results show that after excluding any single firm from the treatment group, rdi remains significantly positive. However, for pac, excluding the three largest firms (Firm B, Firm F, and Firm H) results in pac becoming insignificant. These three firms are recognized as “Little Giant” firms, characterized by significant research and development capabilities and market competitiveness. As a result, they may have received stronger innovation incentives following the implementation of the NDPN policy. Once these firms, with their larger asset sizes and innovation advantages, were excluded, the overall policy response in the sample weakened, leading to the insignificance of the policy effect. This suggests that the NDPN policy’s incentive effect may primarily apply to these leading firms. Future research should further explore the heterogeneity of the policy’s effects across different types of enterprises.

## 4 Discussion

This study analyzed the impact of the NDPN policy on the innovation performance of biopharmaceutical firms in China, using panel data from listed firms in the biopharmaceutical sector from 2018 to 2023. The results indicate that the implementation of the NDPN policy has a significant positive impact on biopharmaceutical firms increasing their innovation investment and innovation R&D output.

The impact of the national drug negotiation policy on the innovation performance of biopharmaceutical firms is primarily achieved through enhancing the accessibility of innovative drugs, shortening the time for innovative drugs to be included in the NRDL, institutional optimizations aimed at “strengthen R&D incentives,” and policy directions that “compel R&D innovation.”

First, the NDPN policy significantly improves the accessibility of innovative drugs, effectively expanding market capacity and thereby stimulating firms’ R&D investment and innovation performance. Multiple empirical studies have shown that (24), after being included in the NRDL, negotiated drugs experience significant price reductions, significantly enhancing patients’ purchasing power, and thereby significantly improving drug accessibility and market scale. Additionally, after the establishment of the “dual-channel” mechanism in 2021, the NDPN policy through the “dual-channel” mechanism and medical insurance fund reimbursement, ensure the effective availability of innovative drugs (25), enabling rapid market expansion of innovative drugs, accelerating revenue generation, and creating a sustainable cash flow cycle, thereby providing a strong financial foundation for continued R&D investment. Furthermore, multiple systematic review studies have indicated that the NDPN policy has improved the accessibility and medical insurance coverage of new drugs (12), effectively promoting the conversion pathway of innovative drug outcomes from approval to clinical implementation; simultaneously, the reimbursement decision-making framework increasingly prioritizes the inclusion of innovative drugs with high clinical value, thereby encouraging firms to focus on the research and development of “high-value” drugs.

Second, the NDPN policy has significantly accelerated the process of innovative drugs being included in medical insurance coverage through institutionalizing a formal price negotiation mechanism. This mechanism has reduced the time it takes for firms to realize returns on their R&D investments, optimized the conversion path for new drugs from approval to clinical use, and effectively improved the innovation efficiency of biopharmaceutical firms. The NDPN policy has significantly shortened the time frame for medical insurance reimbursement decisions. Recent studies indicate that the time from market approval to inclusion in the reimbursement list for innovative drugs in China has been reduced from approximately 5 years to around 1 year, with domestic pharmaceutical firms benefiting more significantly (26). This mechanism has accelerated the market conversion process for new drugs post-market launch and enhanced firms’ R&D enthusiasm. On the other hand, the NDPN policy has significantly improved the utilization rate of innovative drugs. For certain anticancer drugs, both utilization rates and procurement expenditures have seen significant increases following their inclusion in the medical insurance program (35), demonstrating the powerful driving role of medical insurance coverage in promoting the clinical use of medications.

Finally, by aligning institutional policies to better connect corporate innovation and R&D with clinical needs, the NDPN policy has facilitated the transformation of China’s biopharmaceutical industry from an “approval-oriented” model to a “use-oriented” model (27). The clinical entry barriers for innovative drugs into medical insurance have gradually increased, prompting firms to prioritize R&D investments in highly effective new drugs that are likely to gain medical insurance coverage (28). Moreover, the drug evaluation process prior to medical insurance approval has become more stringent, requiring firms to proactively plan the pharmacoeconomic and medical insurance payment suitability of their drugs, which fundamentally drives the shift in R&D logic from an “approval-driven” approach to a “clinical and payment-driven” approach (29).

It should be noted that, in addition to the NDPN policy, the NCVBP was also implemented concurrently and had a significant impact on firms’ innovation investment. Previous studies have shown that the centralized procurement policy significantly reduces the prices of generic drugs, forcing pharmaceutical companies to seek innovative drugs as a strategy to cope with price competition. The intensity of firms’ R&D investment has significantly increased (19), with a more pronounced increase in the R&D investment of firms that were selected (30). Drugs that were not selected face the risk of losing market share. To maintain their market position, companies actively invest in the development of innovative drugs. Moreover, the price compression caused by medical insurance negotiations and centralized procurement forces firms to increase R&D investment to remain competitive. By compressing the profit margins of generic drugs, the centralized procurement policy drives pharmaceutical companies to allocate more resources to innovative R&D, thereby strengthening innovation incentives. In this context, the NDPN and NCVBP policies may interact and jointly drive the innovation strategies of pharmaceutical firms.

This study provides important insights for optimizing the medical insurance access mechanism for innovative drugs. First, a tiered assessment mechanism based on clinical and economic value should be established. Given the continuous growth in the number of innovative drugs in China and the limited availability of medical insurance resources, it is urgent to enhance the efficiency of medical insurance access through a scientifically grounded assessment framework. It is recommended that the medical insurance authorities comprehensively consider therapeutic necessity, clinical effectiveness, and cost-effectiveness of drugs (31), classify innovative drugs into different categories, and set differentiated access procedures and pharmacoeconomic evaluation thresholds accordingly. This mechanism will help prioritize the coverage of drugs with a higher cost-effectiveness ratio within the fund’s affordability range, thereby improving the efficiency of medical insurance fund allocation (32). Second, a risk-sharing mechanism should be introduced to address the potential financial pressure on the fund caused by the inclusion of innovative drugs in medical insurance. Some high-cost drugs may experience a surge in utilization after being covered by medical insurance, which could pose a significant challenge to the medical insurance fund. To mitigate this risk, medical insurance authorities could explore agreements with biopharmaceutical firms to set annual expenditure caps in advance. When costs exceed the pre-set risk threshold, the company would bear part or all of the excess costs. Finally, a dynamic adjustment and re-evaluation mechanism for the NRDL should be established to ensure that medical insurance funds are continuously allocated to high-value drugs. As medical technology advances and the biopharmaceutical market evolves, the NRDL must remain flexible (33). It is recommended to establish a routine re-evaluation system to periodically review drugs already included in the NRDL, focusing on their actual clinical efficacy, scope of use, and funding expenditure. Drugs with proven efficacy and high cost-effectiveness may have their reimbursement scope appropriately expanded, while those with unclear clinical benefits or low usage efficiency should be promptly removed from the directory or have their reimbursement ratios reduced to free up space for truly innovative and high-quality products (34).

This study offers several advantages. First, unlike previous studies that primarily focused on drug pricing or the efficiency of medical insurance expenditures, this study examines the impact pathways of policy interventions on firms’ R&D investment and innovation outcomes from a micro-level perspective, thereby enriching the research scope of medical insurance policy evaluation. Second, this study focuses on the institutional reform of the “negotiation framework” mechanism in the 2019 NDPN, which marked the first attempt to institutionalize and standardize the negotiation process. By combining the significant optimization of negotiation rules and the significant expansion of the sample size of participating drugs, it provides an ideal analytical window with policy shock characteristics. Finally, in terms of methodology, this study employs a combination of DID and PSM-DID identification strategies, supplemented by parallel trend and robustness tests, effectively controlling sample selection bias and unobservable heterogeneity, thereby enhancing the credibility and robustness of the research conclusions.

Nonetheless, this study also has several limitations. First, the sample is limited to biopharmaceutical firms listed on the Shanghai and Shenzhen stock exchanges, excluding unlisted firms that may actively participate in national medical insurance negotiations, and the control group includes firms that applied for the NDPN but were unsuccessful, firms eligible but did not apply, and firms that were ineligible for participation, which may limit the generalizability of the results and introduce bias in the policy effect. However, considering the advantages of listed firms in terms of data availability, industry representativeness, and policy sensitivity, the study still has strong reference value. Second, although this study employed PSM to construct the control group, since the firms that successfully negotiated their medicines for NRDL inclusion are mostly industry leaders, it is therefore difficult to ensure full comparability between the treatment and control groups across all dimensions, which may introduce some selection bias. Finally, during the study period, the government simultaneously implemented multiple policy initiatives, such as the NCVBP policy and medical insurance payment method reforms, which may have confounding effects on firm-level innovation performance. Although this study has made efforts to control this interference through various robustness tests, future research should further explore these issues from the perspective of policy interactions.

## 5 Conclusion

This study utilizes quarterly panel data from 96 listed biopharmaceutical firms in China from the first quarter of 2018 to the fourth quarter of 2023, employing a difference-in-differences (DID) method combined with a propensity score matching (PSM) identification strategy to systematically assess the impact of the NDPN policy on corporate innovation performance. The main findings are as follows: Before the policy was implemented, there were no statistically significant differences in R&D investment and innovation outcomes between firms that participated in the policy and those that did not. However, after the policy was implemented, R&D investment significantly increased among firms that participated in negotiations, and some innovation output indicators also showed positive changes. This suggests that the NDPN policy may have significantly enhanced firms’ innovation momentum by improving drug accessibility, accelerating the timeline for innovative drug market entry, and strengthening incentives for medical insurance coverage. In the future, policymakers can further improve the medical insurance payment mechanism to enhance the efficiency of medical insurance fund utilization while better stimulating the R&D potential and industrial upgrading momentum of biopharmaceutical industry.

## Data Availability

The original contributions presented in the study are included in the article/supplementary material, further inquiries can be directed to the corresponding authors.
